# Vaccine Hesitancy among Healthcare Workers in Europe: A Systematic Review

**DOI:** 10.3390/vaccines11111657

**Published:** 2023-10-28

**Authors:** Mandeep Kaur, Luca Coppeta, Ole F. Olesen

**Affiliations:** 1 European Vaccine Initiative, Universitätsklinikum Heidelberg (Heidelberg University Hospital), Voßstraße 2, 69115 Heidelberg, Germany; ole.olesen@euvaccine.eu; 2 Department of Biomedicine and Prevention, University of Rome Tor Vergata, 00133 Rome, Italy; luca.coppeta@uniroma2.it

**Keywords:** vaccine hesitancy, vaccination rates, healthcare workers (HCWs), Europe

## Abstract

This study analyzes vaccine hesitancy (VH) among healthcare workers (HCWs) in 15 European countries. We have undertaken a systematic review by synthesizing data from 46 articles (between 2015 and 2022) encompassing 55,612 subjects. Despite the heterogeneity of the methods in the various studies, we found that physicians had consistently higher vaccination rates than nurses across different countries and different vaccines. Physicians’ average vaccination rate was 79% across a selection of vaccines, while that of nurses was 62%. Concerns regarding vaccine safety, information gaps, and the responsibility of healthcare authorities in managing VH are highlighted by qualitative insights. This research contributes to our comprehension of the ways in which VH among HCWs is impacted by healthcare roles, vaccine types, and regional disparities. The insights gleaned from this analysis can serve as a guide for targeted interventions aimed at increasing vaccine acceptance and coverage in Europe, ultimately strengthening public health.

## 1. Introduction

Vaccination is one of the most efficient means of infectious disease control and has been successful in preventing infection and lowering mortality rates for many infectious diseases [1]. Despite the scientific and medical consensus on the benefits of vaccination, there is a growing concern about vaccine hesitancy (VH). Vaccine hesitancy is a result of vaccine skepticism, which refers to an individual’s inclination to harbor mistrust or doubt towards vaccines, particularly with respect to their effectiveness, as well as their associated risks and adverse effects [2].

Vaccine confidence varies across nations, vaccination types, and sociodemographic factors, as revealed by previous research [2]. A systematic review of 28 studies revealed that low socioeconomic status is a significant obstacle to the uptake of the human papillomavirus vaccine in Europe [3], while a comprehensive analysis of 20 studies investigating the adoption of measles vaccinations in Europe revealed that vaccine hesitancy is linked to apprehensions regarding adverse effects, a perceived low likelihood of experiencing severe disease progression, skepticism towards healthcare professionals, and advanced age [4]. Similarly, the acceptance of COVID-19 vaccines has been lower in some EU settings than in others, reflecting differences in vaccine confidence and access across the continent [5,6]. According to the “State of Vaccine Confidence in the European Union” report in 2022, overall vaccine confidence across the EU has declined among the general public since 2020 and is now on a similar level as in 2018 [5]. Coverage rates of first-dose measles-containing vaccines have decreased by two percentage points in the European Region of the World Health Organization (WHO) since 2019, with declines exceeding five percentage points in Bulgaria, Malta, and Poland [7]. According to the 2016 global “Survey on Vaccine Confidence”, Europe has seven of the ten least confident countries when it comes to vaccine safety, with 41% of respondents in France and 36% of respondents in Bosnia and Herzegovina disagreeing with the statement that vaccines are safe (compared to the global average of 13%) [8]. The vaccine confidence rate in the general population of the European region ranged from 40.9% to 92.3%, while in healthcare workers (HCWs), it ranged from 54.9% to 95.1% [9].

The vaccination decision-making process is relatively short for some individuals who agree to be vaccinated immediately because they believe it to be the norm, and longer for those who carefully weigh the pros and cons of vaccination by consulting with family, friends, or members of their community; searching the Internet; and consulting with their HCWs. HCWs play an important role in the decision-making process of vaccination as patients rely upon health care providers to learn new health information, advice on vaccine-preventable diseases, and available vaccines [10]. In research conducted in six European nations, the general practitioner (GP), the local pharmacy, and the local hospital were identified as the most reliable providers of health alerts or information on medications [11]. Therefore, healthcare professionals should have in-depth knowledge of the benefits and risks of vaccines, be able to effectively communicate those to patients and their families and be able to address any concerns. At the same time, HCWs are generally at high risk themselves of acquiring or transmitting infectious diseases such as mumps, measles, rubella, Varicella, Hepatitis B, influenza, Pertussis, and COVID-19 [12].

A significant proportion of healthcare workers exhibit a general lack of intention or refusal to receive vaccinations [12,13,14], and similar trends of vaccine hesitancy have been observed in HCWs during the COVID-19 vaccine campaigns [15]. The prevalence of vaccine hesitancy among HCWs varies significantly, however, between different European countries. In Cyprus, only 7% of 436 nurses and midwives were hesitant about receiving the COVID-19 vaccine [16], while a study conducted in France revealed that 23% of 1965 nurses and physicians were vaccine hesitant [17]. These numbers suggest very different perceptions among HCWs in different locations about the underlying concerns or fears regarding the safety and efficacy of vaccines.

Risk perceptions, trust, emotions, beliefs, worldviews, controversies, and crucial events such as outbreaks have a significant impact on HCWs’ attitudes and perceptions about vaccination [18]. The lack of information about vaccines among healthcare workers is another concerning issue: in a recent study about online media and information availability of COVID-19 vaccines, only 10.7% of online information media was targeted towards HCWs, compared to 66.9% of online media that was targeted towards the general population [19]. This implies that HCWs may not have access to sufficiently accurate and up-to-date information about vaccines, which can lead to negative attitudes and perceptions towards vaccination.

The issue of vaccination hesitancy (VH) among healthcare professionals (HCWs) in Europe has become a topic of growing concern in recent years. There have been significant studies carried out in Europe that have examined various elements of vaccination behavior and knowledge among healthcare workers (HCWs). Our research endeavors to offer a unique and complete viewpoint. Previous studies, such as the “HProImmune Survey” [20], which examined immunization-related behavior among HCWs in 14 European countries, and the “Multinational Cross-Sectional Survey” [21] targeting only European medical students and early graduates, have made substantial contributions. These studies provide insights into different dimensions of vaccination attitudes, knowledge, and coverage rates among diverse cohorts within the European healthcare context. Moreover, a qualitative investigation carried out in Croatia, France, Greece, and Romania [22] enabled us to uncover intricate and contextually relevant dimensions of vaccination hesitancy among healthcare workers (HCWs) through an analysis of individuals’ apprehensions over the potential adverse effects of vaccines, their level of confidence in health authorities, and their skepticism towards pharmaceutical corporations. One limitation of this study is its narrow scope since it is only focused on the examination of four countries.

Nevertheless, the uniqueness of our research resides in its specific emphasis on comprehending vaccination hesitancy among healthcare workers (HCWs) throughout Europe, encompassing data from a total of 15 countries. While previous research has examined vaccination attitudes and practices, this study aims to provide a comprehensive analysis of the concerns, uncertainties, and causes connected to the reluctance that healthcare workers (HCWs) may encounter.

Our review focuses on addressing the complex issue of vaccine hesitancy (VH) among healthcare workers (HCWs) in Europe. While previous studies have touched upon aspects of VH, it is important to highlight the specific gaps in the existing knowledge. Previous research has primarily concentrated on single countries or a limited number of European countries, often focusing on specific aspects of vaccine attitudes, knowledge, or practices among HCWs. As a result, there is a dearth of comprehensive, region-wide studies that amalgamate individual research efforts to provide an all-encompassing view of VH among HCWs in Europe. Our research systematically examines all individual studies conducted on and about vaccine hesitancy and immunization behaviors specifically among HCWs across Europe. By synthesizing findings from these studies, our aim is to identify trends, patterns, and shared experiences that transcend national borders. This unique approach is pivotal in providing a holistic understanding of VH within the European HCW community. We have also analyzed cross-country trends of vaccine coverage rates in HCWs per vaccine to better understand the phenomenon of vaccine hesitancy and informed decisions about health policy to better address vaccine hesitancy in HCWs.

## 2. Materials and Methods

### 2.1. Database Search

The search strategy was conducted according to the PRISMA guidelines [23]. We searched the following databases: Google Scholar, PubMed, and Science Direct (Elsevier) for English-language peer-reviewed articles from 2015 to 2022 using a combination of keywords and MeSH terms. Published papers that aimed at evaluating vaccine hesitancy among HCWs in Europe are included in this review. Only studies in the English language that met the inclusion criteria were considered. Inclusion criteria were (1) peer-reviewed published articles; (2) survey studies among health-care workers in Europe; (3) the major aim of the study was to evaluate vaccine hesitancy; (4) publication language was English; and (5) studies focused on European HCWs vaccine hesitancy, its determinants, and HCWs’ willingness for vaccine recommendation, and qualitative and quantitative study methods were used.

The exclusion criteria were (1) unpublished manuscripts (preprints), (2) the article did not aim to evaluate vaccine hesitancy, and (3) publication language was not English. The time period for this search was between December2022 and January2023. The keywords “vaccine”, “vaccination”, “hesitancy”, “HCWs”, “healthcare”, “worker”, “health”, and “professional” were used. The search strategy evolved during the process, and all iterations are provided in Appendix A

**Figure 1:**
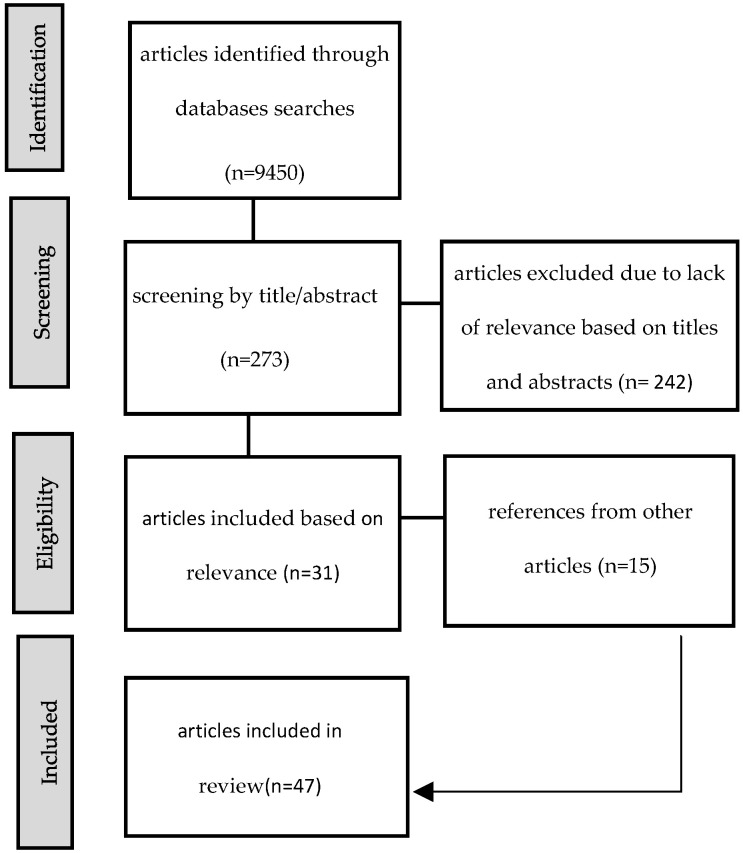
Flow diagram for study selection.

In repeated database queries, the order of keywords was altered to extract the final pool of relevant studies. The database search generated an initial 9450 articles. After removing duplicate titles and articles for which full texts were unavailable, abstracts were manually reviewed according to our inclusion criteria (see Figure 1).

We applied an English language restriction to ensure the feasibility of data extraction and analysis, given the language proficiency of our research team. Additionally, English-language publications are more accessible to our target audience, which includes researchers, policymakers, and healthcare professionals.

### 2.2. Estimation of VH Rates

The research methodologies employed in this study encompass analyzing studies that performed cross-sectional investigations, online anonymous survey surveys, and semi-structured interviews about VH in European HCWs. These studies utilized methods to examine the prevalence and cumulative data on VH, employing both quantitative and qualitative approaches.

**Figure 2:**
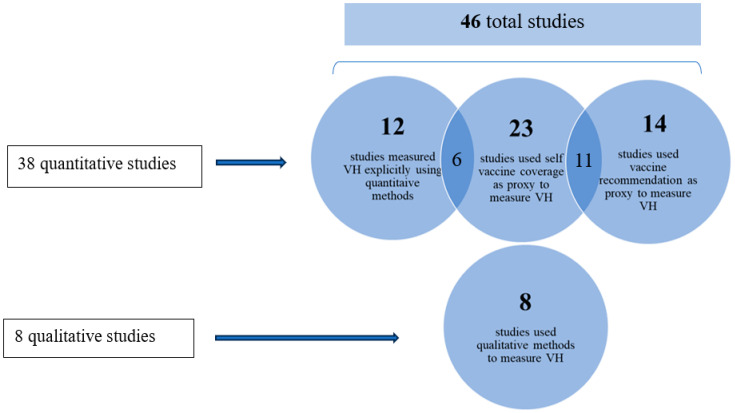
The categorization of different studies according to their methods to measure vaccine hesitancy.

All 46 studies measured vaccine hesitancy; however, out of the total pool of 46 studies, 12 studies [12,16,17,24,25,26,27,28,29,30,31,32] measured vaccine hesitancy explicitly. These studies reported quantitative data on vaccine hesitancy that were used to extract data on VH. Data on VH rates were extracted from studies if participants were “unlikely,” “refused,” “declined,” or “disagreed” to taking a vaccine. Twenty studies used proxies aimed to indirectly assess vaccine hesitancy by analyzing related factors such as attitudes, beliefs, or behaviors. These six studies [23,33,34,35,36,37] measured VH explicitly using proxies, and eight used qualitative methods to gain a deeper understanding of vaccine hesitancy through in-depth interviews and personal narratives (see Figure 2).

### 2.3. Data Extraction

Some types of data extraction were performed automatically via Excel, and data that were missed in the automated transfer were then added manually. For all studies, the same type of information was collected: vaccines considered; population of HCWs sampled; sample size; data collection method; response rate; rates of VH measured (for each HCW group separately); rate and type of VH proxies measured (such as personal vaccination status and/or vaccine recommendations); and data collection period, publication year, journal name, first author, full title of the article, and web address to the article.

A sub-group analysis based on vaccine types was performed. This approach aims to examine the specific factors contributing to vaccine hesitancy in response to different vaccines, considering that healthcare worker (HCW) attitudes may vary based on vaccine characteristics. Data collection was performed by categorizing and analyzing the studies based on the vaccines studied (e.g., COVID-19 vaccines and influenza vaccines) and extracting relevant information accordingly. The mean and median per vaccine was calculated. Random-effects models were used to account for heterogeneity among the included studies.

### 2.4. Risk of Bias Assessment

A risk of bias assessment was added to the manuscript. The domains analyzed were sample size (D1), response rate (D2), and whether VH was measured directly or inferred via proxies (D3). An overall assessment for each study was also performed, and the value of the overall assessment was according to the lowest grade in individual domains. For Domain 1, studies received a ‘low’ risk of bias grade if more than 100 participants were sampled, a ‘some concerns’ grade if the sample size was between 50 and 100, and a ‘high’ concern grade if sample size was under 50. For Domain 2, studies received a ‘low’ risk of bias grade if the response rate was above 40%, a ‘some concerns’ grade if the response rate was between 20 and 40%, and a ‘high’ risk grade if response rates were lower than 20%. If response rates were not published in the study, they received an ‘unclear’ grade. For Domain 3, studies received a ‘Low’ risk of bias grade if they directly measured vaccine hesitancy, for example, by asking participants about their attitudes and beliefs about vaccine safety and effectiveness. Studies received a ‘some concerns’ grade if vaccine hesitancy was not measured directly but inferred through behavioral proxies, which was most commonly the personal vaccination status of participants and the likelihood of recommendations of vaccines to patients (see Appendix B and Appendix C).

## 3. Results

### 3.1. Characteristics of Studies

We identified a total of 46 articles reporting studies of VH in European HCWs (see Table 1). The studies came from 15 different countries, as well as three multi-country studies, and included a total of 55,612 subjects. The study samples varied in size from 10 to 5454 participating HCWs. Two thirds of the total participant number came from just three countries, France, Germany, and the UK. Most studies came from France (12 studies with 15,421 participants), followed by Italy, Germany, and the UK, while eight countries were represented by a single study (e.g., Finland). For other European countries, no studies at all were found. Most studies included general practitioners and/or other physicians (twenty studies with a total of 19,787 participants), followed by nurses (fifteen studies/13,502 participants), other HCWs (nine studies/13,412 participants) (e.g., allied health professionals), healthcare students (five studies/5923 participants), midwives (four studies/985 participants), complementary and alternative medicine (CAM) (two), and dentists (one).

The largest number of studies (22) addressed influenza vaccines, although the largest number of participants were included in the 10 studies on COVID-19 vaccines, despite only having been available since 2020. A relatively large number of studies had also been carried out on the classic childhood vaccines against measles, Diphtheria, Tetanus, and Pertussis, either as individual vaccines or as part of the MMR and DTP combination vaccines (see Table 2).

Out of the 46 studies in the present review, thirty-eight applied quantitative methodologies and gathered data from HCWs through cross-sectional questionnaires [12,16,17,20,21,24,25,26,27,28,29,30,31,32,33,34,35,36,37,38,39,40,42,43,44,45,46,47,49,50,51,52,53,54,56,57,58,59]; eight of the forty-six studies used qualitative methods only to examine data collected from semi-structured interviews or focus groups of HCWs [22,41,48,55,60,61,62,63].

### 3.2. Vaccine Hesitancy among HCWs

Out of the 46 studies in the present review, 18 [12,16,17,24,25,26,27,28,29,30,31,32,33,34,35,36,37,45] explicitly reported a specific percentage value of VH (see Table 3). These studies revealed a huge variation in the prevalence of VH among healthcare workers across different countries and vaccines, with rates ranging from as low as 2.3% among nurses for COVID-19 vaccines [24] to more than 70% among nurses and midwives in Cyprus for COVID-19 vaccines [16].

### 3.3. Estimating VH in HCWs Using Proxy Indicators

Twenty-six out of forty-six studies used proxies to measure VH. Six of the studies measured proxies in addition to explicitly measuring VH, while 20 studies used proxies only. The studies that used proxies are summarized in Table 4. The proxies used to estimate VH included factors such as the self-vaccination status of HCWs (and by extension, their children) [21,46,49,51,52,53,56,57]; negative attitudes or perceptions of vaccination, opinions, and beliefs [20,37,42,43,53,55,58]; vaccine safety and fear of vaccine side effects [31,48]; the frequency of their vaccine recommendations to patients [38,40,43,44,52,61]; the vaccination status of their own children [40,44]; acceptance of vaccine mandates for health-care workers and for children [47,57,59]; their delay/refusal of vaccination for themselves or their children and their patients [34,39,41,50,63]; and vaccine knowledge, confidence, and perceived risks of diseases and vaccines [54,62].

#### 3.3.1. Self-Vaccination of HCWs in Different Countries

Twenty-three of the forty-six studies mentioned “self-vaccination” behaviors of HCWs and used it as an indicator for VH. Self-vaccination refers to the personal vaccination status of HCWs and their children, not necessarily to self-administration of vaccines [20,21,33,34,35,36,37,39,40,42,43,44,45,46,47,49,50,51,52,53,54,56,57,58]. The vaccination coverage (VC) of HCWs collected from different studies and different vaccines is shown below, where the differences in vaccine coverage between physicians and nurses in the same country are compared.

**Figure 3:**
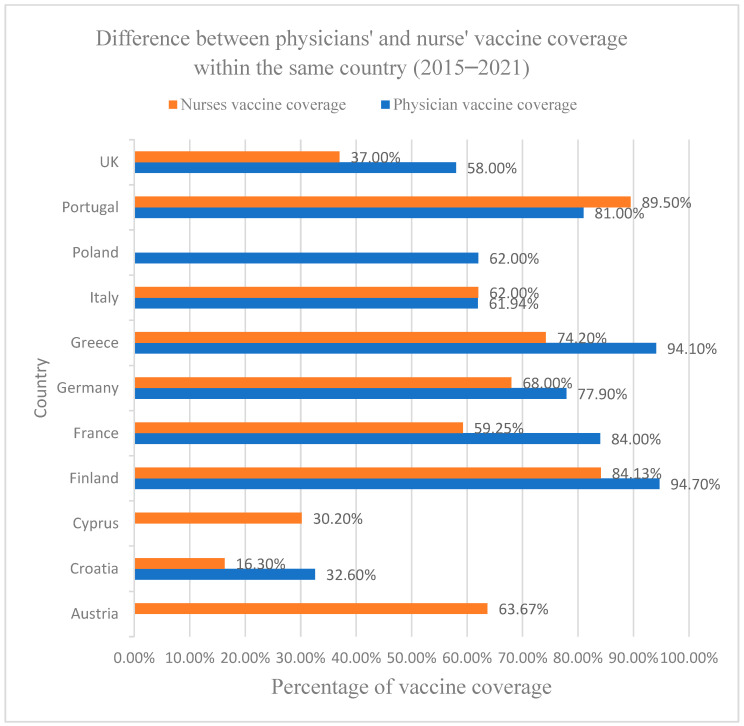
This graph represents the vaccine coverage between physicians and nurses in the same country. It was calculated as the median of all studies on VH in each respective country and contains, therefore, a median of VC of different vaccines. Data on vaccine coverage rates on physicians in Austria and Cyprus and on nurses in Poland are missing, and a comparison between physicians and nurses in these countries is therefore not possible.

The data reveal that physicians, in general, exhibit a higher VC rate in most countries compared to nurses (except in Italy and Portugal), indicating a potential discrepancy in immunization practices between these two crucial healthcare cohorts. The vaccine coverage of physicians ranges from 32.6% in Croatia to over 94% in Finland and Greece, (average 79.1%), while the vaccine coverage of nurses ranges from 16.3% in Croatia to 89.5% in Portugal (average 62.0%). This disparity is attributed to various factors, such as differences in access to vaccines, varying levels of awareness regarding their importance, or diverse beliefs and attitudes towards immunization within the medical community. For instance, a study in Croatia found that 38% of nurses did not receive the influenza vaccine because they believed that they were protected by constant exposure, while 12% were afraid of side effects and 11% believed that vaccines are not effective (see Figure 3).

#### 3.3.2. Vaccine Recommendation by HCWs to Patients and Their Own Children

Fourteen out of forty-six studies in this review analyzed HCWs’ perspectives on vaccine recommendation and used it as a proxy to measure VH [33,36,38,43,44,46,49,50,52,53,56,57,58,59]. The percentage of healthcare professionals who never or occasionally recommended specific vaccines to targeted patients were considered “vaccine hesitant” (see Table 5). Five studies in which nurses were studied along with physicians found that nurses were more hesitant and considered vaccines less safe and beneficial compared to physicians [33,49,50,53,59]. Six studies that studied GPs showed that between 7.3% and 43% of GPs never or occasionally recommended specific vaccines to patients [38,43,44,46,52,58]. Two studies that studied healthcare students found that 12% to 19.4% never or occasionally recommended specific vaccines to patients [36,57].

One study mentioning HCWs’ attitudes toward mandatory vaccination provided no further analysis or interpretation of potential correlations between these attitudes and behaviors; however, they mentioned that actively denying recommended occupational vaccination was more prevalent for influenza (29%) than MMR (7%) [49]. Eighty percent of the students viewed mandatory vaccinations for physicians as an appropriate measure, and 88% would recommend them in settings with immunocompromised patients [57].

A study In Poland reported that 7.8% HCWs either did not recommend vaccines under any circumstances and did not adhere to the childhood immunization program’s recommendations [58], and 19.4% in France did not systematically prescribe all vaccines [36]. In Barcelona, 25% expressed skepticism against a minimum of one vaccine included in the present immunization schedule recommended in Spain. The respondents expressed the greatest skepticism about the Varicella and HPV vaccinations, especially nurses for HBV, HPV, and Pneumococcus [59]. Physicians and nurses in Croatia answered, “I don’t know” (20.9%) and said “no” (14.9%) about recommending the HPV vaccine [33]. In Italy, 98.6% of participants reported recommending the use of the influenza vaccine; in contrast, recommendations for Pneumococcus were reported by 84.9%, while Herpes Zoster recommendations were recorded by 65.6%. Reasons for hesitating in recommending vaccines includes lack of information on the vaccine and doubts on vaccine efficacy [52]. In Finland, observation of recommendation behavior reveals that 13.8% do not offer advice to patients who are hesitant about childhood vaccinations, and 26.1% of HCWs did not recommend the influenza vaccine [50].

### 3.4. Vaccine Coverage for Different Vaccines among HCWs

Looking at the uptake of specific vaccines confirmed the differences between different countries and groups of HCWs.

COVID-19: Six studies addressed VC or acceptance of COVID-19 vaccination [16,17,26,29,32,37]; four of them mentioned the VC of physicians, and five mentioned the VC of nurses. Overall, HCWs in Germany had the highest average vaccination coverage for the COVID-19 vaccine, at 91.7%, followed by Portugal (87%), Italy (82%), Greece (81.9%), France (73.1%), and Cyprus (3%).

Physicians in Greece had a VC of 94.1%; Germany, 92.2%; France, 89.2%; and Portugal, 81.0%. Nurses had a VC rate in Greece of 74.2%, in Germany of 91.0%, in France of 76.2%, and Portugal of 89.5% (higher than the VC of physicians in Portugal), with the lowest in Cyprus at 30.2%.

Across all studies, nurses had a mean average vaccination rate of 72.2% (median: 76.2%), which is significantly lower than the mean average vaccination rate of physicians, which was 89.1% (median: 90.7%). However, the low average is partly influenced by the very low VC from a single study from Cyprus. As only nurses and midwives (included in the “other HCWs” group) were studied, it is unknown if physicians in Cyprus have similarly low VC. When we disregard the study from Cyprus, nurses still have a lower average rate than physicians, with a mean of 82.7% (median: 82.9%), but the difference is less pronounced.

#### 3.4.1. Influenza

Nineteen studies measured VC against seasonal influenza [20,21,25,28,33,35,36,40,42,44,45,46,49,50,51,52,53,54,58], and six of these studies measured VC among different groups of HCWs within the same study, which allows for a direct comparison. Several studies were conducted in the same country, sometimes on the same and sometimes on different HCW groups.

Ten studies from seven different countries revealed that VC for physicians was between 58 and 79%, except in a study in Croatia, which had a vaccination rate of only 32.6% among physicians.

The mean average VC of physicians across all studies was 65.92% (median: 66.1%). There were six cases where physicians were studied in the same country by two independent studies, and VC was generally consistent between the studies.

Nurses were the second most studied group, with eight studies in seven countries. In almost all studies, nurses had a relatively low VC between 16 and 45%, with Finland being an outlier with a vaccination rate of 84.1%. Across all studies, the mean average VC for nurses was 36.2% (median: 32.0%). This cannot be compared to the average physician VC across all studies because nurses and physicians were not usually studied together. In studies that examined both nurses and physicians, nurses had a mean average VC of 40.6%, while physicians had 63.1%.

#### 3.4.2. Hepatitis B (HBV)

There were nine [20,21,35,45,46,51,53,54,57] studies that measured vaccination coverage rates for Hepatitis B among HCWs. It includes three studies from Italy, two from France, one from Germany, and one from Austria—two studies measured coverage rates in multiple countries. Physicians and nurses were the groups most often studied, but only one study in Italy directly compared VC among different HCW groups [51]. In this study, physicians (89.3%) had a VC that was 3.7% lower than nurses (96%). A second study that was conducted in Italy found a similarly high vaccination rate for nurses, at 93% [53]. In France, VC was generally lower, with an 86% vaccination rate for physicians and 61% vaccination rate for nurses [35,45].

#### 3.4.3. Diphtheria, Tetanus, Pertussis (DTP)

There were 12 studies [20,21,35,36,40,42,45,46,51,53,54,57] that measured the vaccine coverage of health care workers for DTP. Four studies focused on physicians: France (2), Italy (1), Germany (1), and VC were found to be between 36 and 88%, with an average of 80.9%. Nurses were also studied, with four studies in three countries (Austria, France, Italy). In almost all studies, nurses had a relatively low VC between 30.7 and 62%, (average 59.7%), with Austria being a notable exception with a vaccination rate of 93.5% among nurses [56]. Across all studies, the mean average vaccination rate for nurses was 60.9% (median: 59.8%). However, this rate cannot directly be compared with the average vaccination rate of physicians across all studies, as VC among nurses and those among physicians were mostly not studied within the same study. There was only a single study from Italy that compared the VC of physicians (36.1%) and nurses (30.7%).

#### 3.4.4. Measles-Containing Vaccines

A total of nine studies [20,21,35,36,49,51,53,54,57] were conducted to measure the VC of healthcare workers (HCWs) for measles-containing vaccines (MMR and measles alone). Two research studies were conducted in Austria, three in Italy, one in the United Kingdom, and two were carried out in several European countries. Physicians were the subject of investigation in a single study in Italy [51], with an average VC of 45.1%, and an “other HCWs” group was examined in five distinct studies. Additionally, nurses were the focus of four studies, students were analyzed in two studies, and midwives were investigated in just one study. The available data on physicians’ vaccination coverage are limited to a single study conducted in Italy [51], which precludes drawing definitive conclusions from the findings.

VC among nurses was between 43.3 and 84%, with a mean of 63.7% (median of 63.8%). According to this study [51], midwives exhibited a vaccination coverage rate (76.90%) that was 28.95% higher than the average rate observed among physicians (45.10%), nurses (43.30%), and other healthcare workers (26.50%). Two studies conducted in Austria and across Europe reported VC among students with rates of 78% and 81.5%, respectively. However, as no other healthcare groups were included in these studies, no definitive conclusions or comparisons can be drawn [21,44].

### 3.5. Qualitative Studies

Our review included eight qualitative studies [22,41,48,55,60,61,62,63] that offered insightful information on VH among HCWs. They described health care workers’ VH-related concerns and their interactions with hesitant patients [22], their reasons for VH [41], and CAMs vaccine-related experiences and behaviors [61]. The link between vaccination side effects and vaccine hesitancy among German healthcare workers (HCWs) [48], as well as their perceptions of their role in vaccination recommendation, were analyzed [63], and another study on complementary and alternative medicine (CAM) practitioners investigated the beliefs and opinions among vaccine-hesitant doctors in Austria [55,61]; one study investigated the reasons for non-vaccinated nursing staff to decline seasonal influenza vaccination [62].

GPs in a multi-country European study (Croatia, France, Greece, and Romania) expressed concerns about inadequate information about vaccine safety and overexposure risks [31]. Wilson et al. (2020) [41] revealed that health scandals and vaccine controversies, which participants felt were poorly managed by health authorities, ruptured the previously established unspoken agreement between health authorities and GPs. The rupture of this implicit agreement has resulted in a breakdown of trust not only in health authorities but also in the vaccine recommendations. Two studies were conducted in Switzerland [61] and Austria [55] on doctors practicing or supporting CAM. These practitioners exhibited diverse perspectives on vaccination, expressing mixed attitudes towards vaccines. They stressed the importance of customized vaccination strategies by evaluating each vaccine on an individual basis [55,61]. They also expressed doubts about the reliability of vaccine studies [55] and uncertainty regarding potential long-term effects on children’s immune systems, particularly concerning autoimmune diseases [61]. Despite official guidelines suggesting the first vaccination at 2 months, providers frequently postponed vaccines until 6 months or older. Some doctors did not recommend specific vaccines, such as MMR, Polio, HBV, or HPV, deviating from standard recommendations. All CAM participants in Austria advocated for a delayed administration of the measles vaccine [55]. The study by Holzmann-Littig et al. (2019) [48] aimed to understand the specific short-term and long-term side effects that cause HCWs to worry and hesitate to get vaccinated. The most feared short-term adverse effects were vaccination reactions, allergic reactions, and daily limitations. The most feared long-term effects were auto-immune reactions, neurological adverse effects, and presently unknown long-term effects. A study in the Czech Republic calculated the positive vaccination perception rate, or PVPR, between students enrolled in teacher education program and medical students. Medical students’ vaccination acceptance rate was 92%, while teacher education students was 72%, which were studied in the same study [60].

A negative attitude towards vaccines was also reflected in a study conducted in the Netherlands, where GPs questioned the efficacy of the influenza vaccination. Some general practitioners disagree with the notion of immunizing elderly patients against illnesses other than Tetanus and influenza [63].

In one study [62], investigators sought to understand the reasons behind the low seasonal influenza vaccination rates among non-vaccinated nursing staff. The analysis illuminated three interconnected themes that elucidated the reasons behind nurses’ refusal of influenza vaccination. Firstly, the notion of maintaining a strong and healthy body emerged as a central factor influencing their decision to reject the vaccine. Secondly, the nurses expressed a desire to uphold their decision-making autonomy, particularly concerning their bodies and well-being. Thirdly, they felt as though their goal of maintaining a strong and healthy body was at odds with an environment that they believed to be unreliable. The nurses believed that maintaining a strong and healthy body was crucial not only for their own well-being but also for the care they provide to their patients. They were concerned that the influenza vaccine might introduce unknown substances into their bodies, potentially compromising their overall health. Additionally, they expressed skepticism towards the reliability of the vaccine’s effectiveness, citing instances where they had seen colleagues fall ill despite being vaccinated. This lack of trust in the vaccine’s efficacy further reinforced their decision to refuse it.

### 3.6. Knowledge, Beliefs, and Concerns about Vaccines

HCWs’ knowledge, beliefs, and vaccine-related concerns, reasons for VH, experiences, and behaviors were reported in various studies and were similar to those of the general public. Vaccine hesitancy was linked to a fear of vaccine-related adverse side effects [16,17,26,30,33,37,39], lack of information about new vaccines [26], influence from people in their personal environment who refused the vaccine [48], and reduced trust in vaccines [60]. Vaccine recommendation was also connected with safety concerns; GPs were less likely to prescribe a vaccine if they had safety worries [48,52]. Several studies [17,29,30,35,36,38,39,41,43,50,52,55,56] found a lack of trust in medical/pharmaceutical institutions and attitudes correlated with VH. Receiving information advocating against vaccination from family members rather than HCWs or government websites was associated with increased hesitancy towards regular COVID-19 vaccination [30]. Although, overall, nurses and other healthcare professionals were generally found to have higher levels of VH than physicians, we found no definitive answer as to why nurses in Europe have more vaccine hesitancy than physicians. In some cases, the vaccine coverage of some HCW groups was higher than that of physicians; e.g., midwives were more likely to be vaccinated against measles compared to physicians [51]. Nursing coordinators (57%) were more likely to be vaccinated than nurses (18%) against influenza [53]. The type of profession or health education programs was highly correlated with VH in several studies [31,33,35,41,50]. Doctors who practiced or supported complementary and alternative medicine (CAM) focused on individualized vaccination strategies and were uncertain about the risk of long-term side effects. They emphasized the importance of respect, empathy, and patient involvement in vaccination decisions [55,61].

## 4. Limitations

This present review has several limitations. This review only includes studies from 15 countries in Europe. Only studies in English were included, and relevant studies in other languages might have therefore been omitted. Additionally, this review only focused on studies that were published in peer-reviewed journals, which may have left out valuable information from non-peer-reviewed or institutional sources. Considering a variety of vaccines, we assessed the prevalence of VH among healthcare workers, in general. We did not use a standard instrument to evaluate the quality of the studies, but we did systematically identify each study’s methodological strengths and limitations. It is crucial to acknowledge that varying studies have employed distinct definitions and metrics for VH, potentially affecting the comparability of our findings.

The heterogeneity of the methodological approaches used to assess VH in the quantitative studies made it difficult to compare their results. Few studies assessed VH prevalence explicitly in HCWs, and many studies relied on proxy indicators. Proxy indicators consisting of behavioral indicators, such as self-immunization or vaccination recommendations, were used to measure VH. Some studies used scores to measure VH levels, which had the benefit of allowing the degree of VH to be measured [33,36]. Most of the studies (38 out of 46) used online questionnaires, and low response rates may limit representativeness in some cases. Moreover, the observed heterogeneity in the analysis strategies makes it difficult to quantify the relative importance of the various determinants of HCWs’ VH, even though this is essential for defining an intervention. Overall, while this review provides valuable insights into the prevalence of VH in HCWs in Europe, it is important to approach these findings with an understanding of their limitations and potential biases.

## 5. Discussion

Vaccine hesitancy among HCWs is complex and multidimensional. It is important to identify and design targeted interventions to combat it. In this review, we found that vaccine hesitancy does exist among healthcare workers in Europe, and it exhibited significant variation across different countries and vaccines.

Factors Affecting Hesitancy: The WHO 5C model of VH, i.e., confidence, complacency, constraints (adjustment of the term ‘convenience’ to now include both structural and psychological barriers), calculation (preference for deliberation), and collective responsibility (communal orientation), appears to fit well as a first explanatory model for VH in HCWs [64]. The underlying factors contributing to vaccination hesitancy among healthcare workers (HCWs) exhibit similarities to those reported in the general public. It is influenced by the presence of public conflicts, the effect of media coverage, and the dynamics of encounters with patients who express hesitancy [31,38,40,44,56]. Factors included a lack of trust in institutions; fear of side effects, such as a lack of confidence in vaccines, lack of knowledge, perceptions or beliefs about vaccines, and perceived low risks of VPDs; cultural and religious influences; socioeconomic status; access to healthcare services; and health literacy [21,22,38,39,41,56]. This lack of trust can vary in intensity depending on the cultural and sociopolitical context. HCWs who lack trust in institutions are more likely to be vaccine-hesitant and less likely to recommend vaccines to their patients. The absence of trust can be ascribed to several issues, such as the perceived insufficiency of support for public health immunization obligations and the presence of conflicts of interest between health authorities and pharmaceutical corporations [22,65,66].

Furthermore, healthcare workers (HCWs) who engage in the practice of complementary and alternative medicine (CAM) tend to have a greater inclination towards adopting a critical perspective on conventional health systems [27,46,47,55]. Vaccine coverage information only concerns physicians and nurses, leaving out other healthcare workers who also play a significant role in administering vaccines. There is a clear indication that there exists a want for enhanced education and training pertaining to vaccinations, since the distribution of information for healthcare workers often exhibits fragmentation, emanating from many sources.

Impact on Public Health: The ramifications of vaccination hesitancy among healthcare workers (HCWs) are significant and have far-reaching repercussions for public health. Healthcare workers (HCWs) play a crucial role in both obtaining vaccinations themselves and in providing recommendations and giving vaccines to patients. When healthcare workers (HCWs) exhibit hesitancy or lack of sufficient knowledge, it might lead to a decrease in vaccination rates throughout the broader population. Consequently, this presents considerable obstacles in the pursuit of herd immunity and the protection of susceptible demographics.

Recommendations: In a cross-country comparison for various vaccines, we found that nurses were generally more vaccine hesitant than physicians. Why nurses are more hesitant than physicians is a complex issue and can be influenced by various factors. Additional research is required to determine the underlying causes of this disparity in vaccination rates among healthcare professionals.

There were also exceptions reported to the general trend of physicians having higher vaccination coverage. Physicians were above average in influenza vaccination rates, but nurses had the highest Hepatitis B vaccine coverage rates, followed by midwives and physicians. For DTP and measles, midwives had higher vaccination rates than physicians. These results suggest that healthcare workers’ attitudes and beliefs about vaccines may differ depending on the type of vaccine, and that tailored interventions may be required to increase vaccination rates across various healthcare professions. Notably, these results are based on self-reported information and may not reflect actual vaccination rates.

It is important for healthcare organizations and governments to address these concerns through targeted education and communication efforts. These initiatives should ensure that all HCWs are protected against vaccine-preventable diseases and can continue to provide essential care to patients. Additionally evaluating and enhancing immunization courses in the initial training programs of HCWs is vital. Furthermore, it is essential to broaden the scope of vaccination rate assessments to encompass all HCWs, as each play a significant role in administering vaccines.

The degree and frequency of reluctance of VH in HCWs may differ, but it continues to be a noteworthy issue. The implementation of customized treatments and methods is of utmost importance in order to enhance vaccine acceptability, protect public health, and alleviate the impact of vaccine-preventable illnesses in Europe. To provide the best possible care for their patients, all healthcare professionals must be knowledgeable and receive current information on vaccine recommendations and administration.

## 6. Conclusions

Vaccine hesitancy is not confined to the general public but extends to HCWs across Europe. The prevalence and intensity of VH varies across vaccines, countries, regions, professions, and sociodemographic factors. Vaccine coverage was higher in countries such as France, Germany, and Finland compared to other European countries.

It is crucial to acknowledge that vaccination hesitancy among healthcare professionals, while often more prevalent among nurses than physicians, is influenced by multifaceted factors. These encompass a fear of side effects and a deficit in confidence towards health regulatory bodies, the pharmaceutical sector, and government institutions. Our findings emphasize that European healthcare workers’ vaccine hesitancy poses a significant threat to public health by undermining vaccination recommendations.

Despite the substantial insights gained from our review, it is essential to acknowledge the limitations. The lack of a universal definition for vaccine hesitancy and the absence of comparable data across various European countries and healthcare worker groups make it challenging to precisely gauge the extent of hesitancy.

In order to effectively mitigate healthcare worker reluctance, it is imperative to implement customized treatments and methods that specifically target the underlying reasons leading to this issue. These endeavors will not only enhance the acceptance of vaccines but also provide valuable contributions to the efficacy of vaccination programs, safeguarding susceptible populations, and alleviating the impact of vaccine-preventable illnesses in Europe. It is important to cultivate an atmosphere characterized by trust, knowledge, and accessibility in order to facilitate the acquisition of information and skills by healthcare practitioners, so enabling them to make well-informed decisions and deliver optimal care to their patients. By acknowledging and resolving these problems, we assume a crucial role in protecting and promoting public health, as well as advocating for the effectiveness of immunization programs.

## Figures and Tables

**Table 1:** No. of studies per country and respective sample size, ordered according to total sample size for each country. Sample size is the total number of participants in all studies in a specific country. Three studies covered HCWs from several countries, summarized under “Europe”.

Country	Total Sample Size	No. of Studies	References
France	15,421	12	[12,17,35,36,38,39,40,41,42,43,44,45]
Germany	12,606	5	[28,29,46,47,48]
United Kingdom	9587	4	[25,30,31,49]
Europe	7310	3	[20,21,22]
Finland	2962	1	[50]
Italy	2136	7	[24,26,27,51,52,53,54]
Austria	1312	3	[55,56,57]
Greece	1282	1	[32]
Portugal	890	1	[37]
Poland	500	1	[58]
Cyprus	437	1	[16]
Spain	414	2	[34,59]
Czech Republic	386	1	[60]
Croatia	324	1	[33]
Switzerland	35	2	[61,62]
The Netherlands	10	1	[63]
Total	55,612	46	

**Table 2:** No. of studies per vaccine and number of participants in the studies for each vaccine. Several studies studied multiple vaccines at the same time.

Vaccines	No. of Studies	Total Sample Size
Influenza vaccines	22	22,499
Measles-containing vaccines ^1^	16	22,320
HBV (Hepatitis B)	14	17,398
DTP (Diphtheria, Tetanus, and Pertussis)	14	16,327
Vaccination hesitancy in general	12	7727
COVID-19 vaccines	10	23,215
Varicella vaccines	10	9416
HPV (Human Papillomavirus)	9	6900
MenC (Meningococcal C)	9	12,068
Pneumonia vaccines	8	9661
HAV (Hepatitis A)	5	7107
Tuberculosis vaccines	3	6080

^1^ This total includes fourteen studies that covered MMR (measles, mumps, and rubella) vaccines and two studies that covered measles vaccines.

**Table 3:** Summary of results on VH prevalence of different studies ^1^.

Vaccines	Country	Sample Size	VH Prevalence N (%)	Study Population	Study	Study No.
COVID-19	Cyprus	436	305 (70%) VH	nurses, midwives	Fakonti et al., 2021	[16]
	France	1965	453 (23%) VH, 76 (4%) reluctant	nurses, physicians	Paris et al., 2021	[17]
	Italy	531	12 (2.3%) VH, 33 (6.2%) undecided	nurses	Aurilio M et al., 2021	[24]
	Italy	421	75 (17.8%) VH	dentists	Belingheri et al., 2021	[26]
	Germany	4500	167 (3.7%) undecided, 208 (4.6%) openly refused	HCWs ^5^	Christopher Holzmann-Littig et al. 2021	[29]
	United Kingdom	5454	1282 (23.5%) VH	HCWs ^2^	Neyme Veli et al., 2020	[30]
	United Kingdom	3235	862 (26.7%)	HCWs ^3^	Katherine Woolf et al., 2022	[31]
	Portugal	890 ^6^	56 (19%)	physicians	Estrela et al., 2022	[37]
			28 (10.5%)	nurses		
			18 (7.3%)	pharmacists		
			11 (13.6%)	dentists		
	Greece	1309	237 (18.1%) declined vaccines	HCWs ^4^	Mitsikostas et al., 2021	[32]
VH in general	France	1795	718 (40%) moderate VH, −14 (0.8%) high VH	physicians	Verger et al., 2021	[12]
	Italy	108	9 (8%) unvaccinated ^7^	HCWs ^8^	Paoli et al., 2019	[27]
	France	542	108 (20%) (score ≥ 3)	healthcare students	Baldolli et al., 2020	[36]
VH in general, influenza, HPV, MMR	France	1712	166 (11%) moderately VH, 56 (3%) highly VH	general practitioners	Verger et al., 2016	[45]
	Croatia	324	55 (17%) VH (score ≤ 81)	general practitioners, paediatricians, nurses	Tomljenovic et al., 2021	[33]
Influenza	United Kingdom	765	209 (27%) declined	HCWs ^9^	Lewthwaite P et al. 2014	[25]
	Germany	677	20 (3%) VH	physicians, nurses	Hagemeister et al., 2018	[28]
Childhood vaccines ^10^	Spain	137	44 (32.1%) VH	paediatric health professionals	Elizondo-Alzola et. al, 2021	[34]
Mandatory and recommended vaccines for HCWs ^11^	France	1539	670 (44%) VH	nurses	Wilson et al., 2020	[35]

^1^ Percentages on VH cannot be easily compared between different studies, as each study used a different set of criteria on what attitudes and behaviors constitute VH or had different delineations between the less strong “vaccine reluctancy” and “vaccine hesitancy”. Some studies simply inquired on vaccination status, with unvaccinated participants being automatically categorized as VH. Other studies determined VH status through a questionnaire on attitudes and beliefs, through which vaccinated people could also be classified as VH. These studies also often employed a scoring system, and participants could, therefore, have higher or lower VH scores; ^2^ e.g., doctors, nurses, allied health professionals, and others; ^3^ medical, nursing (inc. midwives + health care assistants), AHPs (not inc. scientists), pharmacists, healthcare scientist, ambulance, dental, optical, admin/estates/other, and missing [22]; ^4^ physicians, nurses, physiotherapists, social workers, psychologists, and biologists; ^5^ certified nurses, other non-physician medical staff, residents, physicians with specialist/personnel responsibility, administration/science, dentist/dentistry student/dental assisting personnel, and others. ^6^ Physicians = 294, nurses = 267, pharmacists = 248, dentists = 81. ^7^ The unvaccinated respondents answered that they do not consider themselves at risk (25%), reported never getting sick (21%), and think that influenza is not a serious disease (31%) [19]; ^8^ e.g., doctors, nurses, allied health professionals, and others ^9^; senior doctors, junior doctors, nurses, professions allied to medicine (PAM), including physiotherapists, occupational therapists, and so forth; and healthcare assistants (HCAs). ^10^ Diphtheria, Tetanus, Whooping cough, Polio, H. Influenzae, Hepatitis B, Meningococcus C, Hepatitis A, measles, rubella, Parotitis, HPV, Varicella, Pneumococcus (46). ^11^ Mandatory BCG, DTP, HBV; recommended: influenza, Pertussis, Varicella, and MMR.

**Table 4:** Proxies used to measure vaccine hesitancy.

Proxies	Study No.
Self-vaccination of HCWs	[20,21,33,34,35,36,37,39,40,42,43,44,45,46,47,49,50,51,52,53,54,56,58]
Vaccine recommendation by HCWs to patients and their own children	[33,36,38,43,44,46,49,50,52,53,56,57,58,59]

**Table 5:** Representation of studies that used vaccine recommendations as a proxy to vaccine hesitancy.

Vaccines	Country	Sample Size	Did Not Recommend Vaccines	Study Population	Study	Study No.
HBV, HPV, influenza, MenC, MMR	France	1712	16%	GPs	Verger et.al., 2015	[38]
HPV, influenza, MMR	Croatia	324	20.9% don’t know. 14.9% no	GPs and Nurses	Tomljenovic et.al., 2021	[33]
Influenza, HBV, Pertussis	Germany	700	- ^3^	GPs	Neufeind et.al., 2020	[46]
Vaccination hesitancy in general	France	542	19.4%	Healthcare Students	Baldolli et.al., 2020	[36]
Vaccination hesitancy in general	Poland	500	7.8%	GPs and Paediatricians	Stefanoff et.al., 2020	[58]
Influenza, Varicella, Pneumonia	Italy	73	- ^4^	GPs	Vezzosi et.al., 2019	[52]
HBV, HPV, influenza, MenC, MMR	France	2586	29% in NW 25% in CW- ^5^	GPs	Collange et.al., 2019	[43]
DTP, HAV, HBV, influenza, MenC, MMR, Pneumonia, Tuberculosis, Varicella	Italy	85	35%	Nurses and nursing coordinators	Tamburrano et.al., 2019	[53]
Childhood vaccinations ^6^	Finland	2962	13.8%	GPs, nurses, head nurses	Karlsson et.al., 2019	[50]
Influenza, MMR	United Kingdom	133	- ^7^	Medical nurses, administrative staff, midwives	Little et.al., 2015	[49]
Childhood vaccinations ^1^	Spain	277	25% ^2^	Paediatricians and Nurses	Picchio et.al., 2019	[59]
HBV, HPV, MenC, MMR	France	1038	43%	GPs	Agrinier et.al., 2017	[44]
HAV, HBV, influenza, MMR, Pertussis, Varicella	Austria	1184	12%	Medical students	Kunze et.al., 2020	[57]

^1^ (Diphtheria, Hepatitis A, Hepatitis B, HPV, measles, MenC, Parotitis, Pneumococcus, Polio, influenza, rubella, Tetanus, Varicella, Whooping cough) [21]. ^2^ Skeptical about recommended childhood vaccines in Spain [21]. ^3^ Majority claimed to recommend vaccinations to patients; the study did not specify the number [46]. ^4^ A total of 98.6% reported recommending the influenza vaccine, recommendations for the Pneumococcus vaccine were reported by 84.9%, while Herpes Zoster recommendations were recorded by 65.6% of participants. ^5^ GPs’ scores for vaccine recommendation frequency and trust in official sources; vaccination recommendations were lower in northwest (29%) and midwest France (25%) than in the rest of France (34%, *p* < 0.05) [43]. ^6^ Childhood vaccinations (rotavirus vaccine, Pneumococcal conjugate vaccine (PCV) against meningitis, pneumonia, sepsis, and ear infection), the DtaP-IPV-Hib vaccine (the ”5-in-1 vaccine” against Diphtheria, Tetanus, Pertussis, Polio, and Hib diseases, such as meningitis, epiglottitis, and sepsis), the MMR vaccine (against measles, mumps, and rubella), and influenza (self-vaccination). ^7^ Provided no further analysis or interpretation; however, mentioned denial for influenza (29%) was higher than MMR (7%) [49].

**Figure A1:**
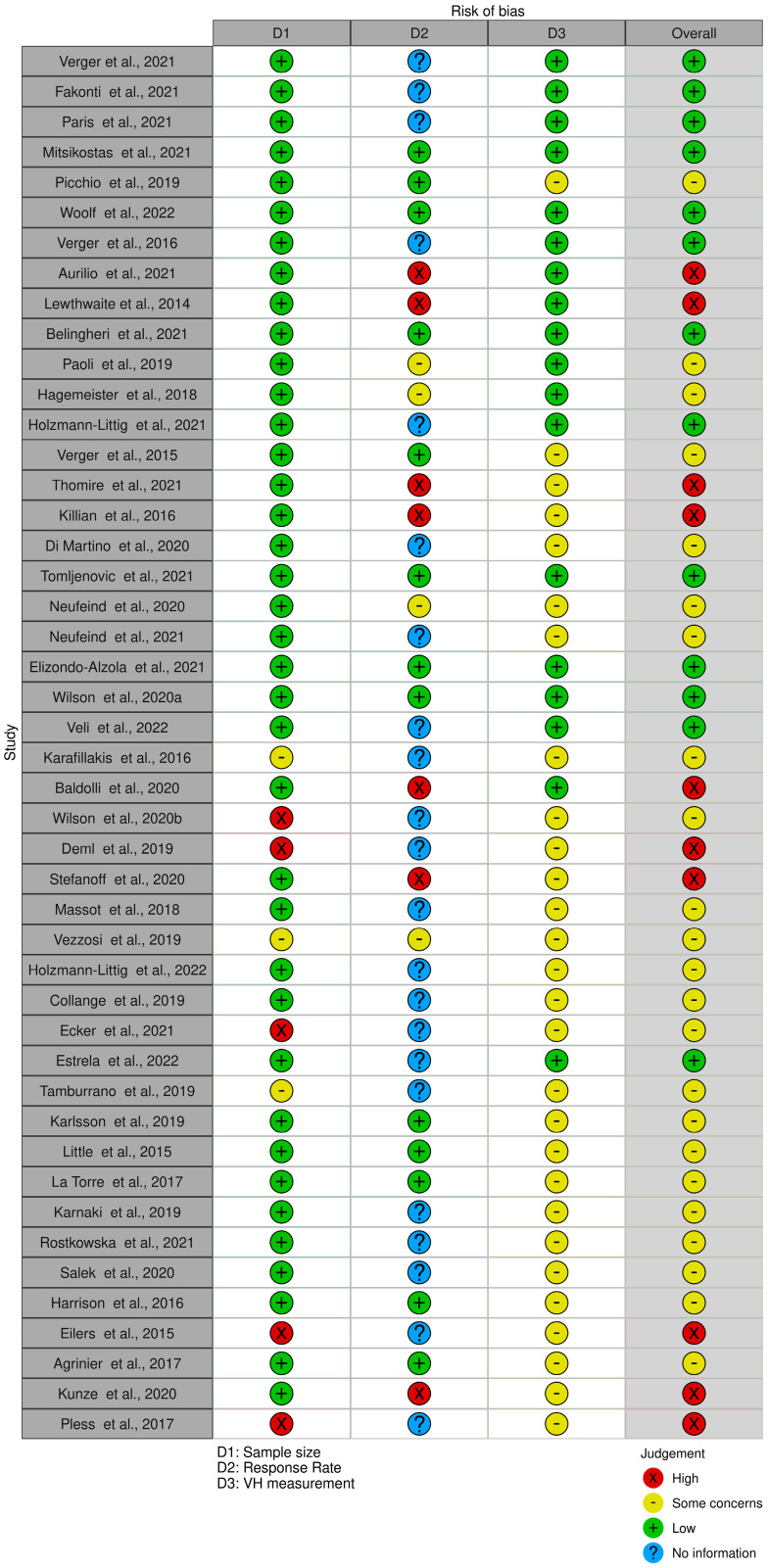
Risk of bias assessment ([12,16,17,20,21,22,24,25,26,27,28,29,30,31,32,33,34,35,36,37,38,39,40,41,42,43,44,45,46,47,48,49,50,51,52,53,54,55,56,57,58,59,60,61,62,63]).

**Figure A2:**
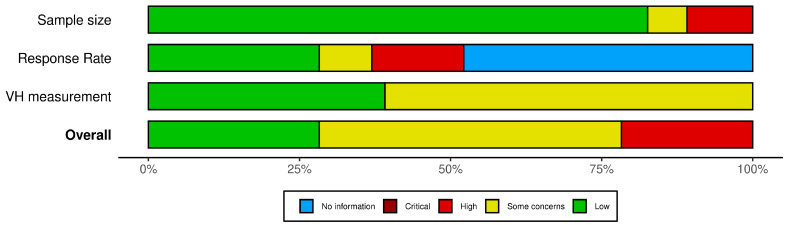
Risk of bias summary.

## Data Availability

The datasets generated and analyzed during this current study are available from the corresponding author upon reasonable request.

## Abbreviations

CAM	Complementary and Alternative Medicine
DTP	Diphtheria, Tetanus, and Pertussis
GPs	General Practitioners
HAV	Hepatitis A
HBV	Hepatitis B
HCWs	Health Care Workers
HPV	Human Papillomavirus
MenC	Meningococcal C
MMR	Measles, mumps, and rubella
NA	Not Applicable
TB	Tuberculosis
VC	Vaccine Coverage
VH	Vaccine Hesitancy
VPDs	Vaccine-Preventable Diseases

## Appendix A

**Table A1:** Search strategy.

Search Component	Keywords/Phrases
Population	Healthcare workers, medical staff, nurses, physicians, doctors, hospital staff, healthcare professionals, HCWs “Healthcare workers” OR “Medical staff” OR “Nurses” OR “Physicians” OR “Doctors” OR “Hospital staff” OR “Healthcare professionals”
Vaccine Hesitancy	Vaccine hesitancy, vaccine refusal, vaccine acceptance, vaccination attitudes, vaccine beliefs, vaccine concerns, vaccine confidence “Vaccine hesitancy” OR “Vaccine refusal” OR “Vaccine acceptance” OR “Vaccination attitudes” OR “Vaccine beliefs” OR “Vaccine concerns” OR “Vaccine confidence”
Europe	Europe, European countries, European Union, EU, Western Europe, Eastern Europe, Northern Europe, Southern Europe, Central Europe (“Europe” OR “European countries” OR “European Union” OR “EU” OR “Western Europe” OR “Eastern Europe” OR “Northern Europe” OR “Southern Europe” OR “Central Europe” OR “Austria”, OR “Belgium”, OR “Bulgaria”, OR “Croatia”, OR “Republic of Cyprus”, OR “Czech Republic”, OR “Denmark”, OR “Estonia”, OR “Finland”, OR “France”, OR “Germany”, OR “Greece”, OR “Hungary”, OR “Ireland”, OR “Italy”, OR “Latvia”, OR “Lithuania”, OR “Luxembourg”, OR “Malta”, OR “Netherlands”, OR “Poland”, OR “Portugal”, OR “Romania”, OR “Slovakia”, OR “Slovenia, OR “Spain” OR “Sweden”, OR “Switzerland”, OR “United Kingdom”)
Research Type	Study types: systematic review, meta-analysis, observational study, cross-sectional study, cohort study, qualitative study, survey, qualitative study
Language Restriction	English language
